# Septins and K63 ubiquitin chains are present in separate bacterial microdomains during autophagy of entrapped *Shigella*

**DOI:** 10.1242/jcs.261139

**Published:** 2023-04-13

**Authors:** Damián Lobato-Márquez, José Javier Conesa, Ana Teresa López-Jiménez, Michael E. Divine, Jonathan N. Pruneda, Serge Mostowy

**Affiliations:** ^1^ Department of Infection Biology, London School of Hygiene and Tropical Medicine, Keppel Street, London WC1E 7HT, UK; ^2^ MISTRAL beamline, ALBA Synchrotron Light Source, Cerdanyola del Vallès, 08290 Barcelona, Spain; ^3^ Department of Molecular Microbiology & Immunology, Oregon Health & Science University, Portland, OR 97239, USA

**Keywords:** Autophagy, Cytoskeleton, Cryo-SXT, Septin, *Shigella*, Ubiquitin

## Abstract

During host cell invasion, *Shigella* escapes to the cytosol and polymerizes actin for cell-to-cell spread. To restrict cell-to-cell spread, host cells employ cell-autonomous immune responses including antibacterial autophagy and septin cage entrapment. How septins interact with the autophagy process to target *Shigella* for destruction is poorly understood. Here, we employed a correlative light and cryo-soft X-ray tomography (cryo-SXT) pipeline to study *Shigella* septin cage entrapment in its near-native state. Quantitative cryo-SXT showed that *Shigella* fragments mitochondria and enabled visualization of X-ray-dense structures (∼30 nm resolution) surrounding *Shigella* entrapped in septin cages. Using Airyscan confocal microscopy, we observed lysine 63 (K63)-linked ubiquitin chains decorating septin-cage-entrapped *Shigella*. Remarkably, septins and K63 chains are present in separate bacterial microdomains, indicating they are recruited separately during antibacterial autophagy. Cryo-SXT and live-cell imaging revealed an interaction between septins and LC3B-positive membranes during autophagy of *Shigella*. Together, these findings demonstrate how septin-caged *Shigella* are targeted for autophagy and provide fundamental insights into autophagy–cytoskeleton interactions.

## Teaser

**Summary:** Our combination of super resolution microscopy and cryo-soft X ray tomography provides fundamental insights into how septin cage entrapment of *Shigella* is coordinated with ubiquitin-mediated autophagy.

## INTRODUCTION

Septins are an evolutionarily conserved family of GTP-binding proteins that interact with cellular membranes to form nonpolar filaments and higher-order ring-like structures (Spiliotis and McMurray, 2020; Woods and Gladfelter, 2020). Septin interactions with the plasma membrane underpin a variety of eukaryotic cell hallmarks, including the cytokinetic furrow, cellular protrusions (e.g. cilium and dendritic spines) and the phagocytic cup surrounding invasive bacterial pathogens (Lobato-Márquez and Mostowy, 2016; Mostowy and Cossart, 2012). In the cytosol, septins can entrap *Shigella flexneri* in cage-like structures to restrict actin-based motility (Mostowy et al., 2010), and mitochondria can promote this process (Sirianni et al., 2016). To counteract septin cage entrapment, actin-polymerizing *S. flexneri* can fragment mitochondria (Sirianni et al., 2016). Our previous work has shown that septins recognize micron-scale curvature presented by bacterial membranes enriched in cardiolipin (Krokowski et al., 2018). More recently, we developed an *in vitro* reconstitution assay using purified proteins to dissect the mechanisms underlying septin recognition of growing bacterial cells (Lobato-Márquez et al., 2021). Despite recent insights, the role of septin interactions with host cell membrane during *S. flexneri* cage entrapment is poorly understood.

In addition to restriction of actin-based motility, septin cages target entrapped bacteria for destruction by autophagy (Krokowski et al., 2018; Mostowy et al., 2010; Sirianni et al., 2016). Autophagy, an evolutionarily conserved degradative process that breaks down cytosolic material inside double-membrane vesicles (autophagosomes) after fusion with lysosomes, has key roles in maintaining cellular homeostasis, recycling damaged organelles and providing nutrients during starvation (Bento et al., 2016; Dikic and Elazar, 2018). Selective autophagy is an important host defense mechanism that recognizes intracellular bacterial pathogens for degradation (also called xenophagy) (Grumati and Dikic, 2018). The best-described mechanism underlying selective autophagy is via ubiquitylation of host or bacterial proteins (Randow et al., 2013). Ubiquitylation is a highly versatile posttranslational modification regulating a wide variety of fundamental cellular processes. Ubiquitin is a small protein (76 amino acids) that contains seven lysine residues and an N-terminal methionine residue that can be attached to another ubiquitin monomer (Hershko and Ciechanover, 1998); in this way, proteins can be modified with different polyubiquitin lengths and linkages that direct distinct signaling outcomes (Yau and Rape, 2016). In the case of xenophagy, components of *Salmonella enterica* subsp. *enterica* serovar Typhimurium and *Mycobacterium tuberculosis*-containing phagosomes are targeted with lysine 63 (K63)-linked ubiquitin chains (Fiskin et al., 2016; Manzanillo et al., 2013; van Wijk et al., 2012). Autophagy adaptor proteins [such as p62 (also known as SQSTM1), NDP52 (CALCOCO2) and OPTN] can bind ubiquitin and recruit microtubule-associated protein light chain 3B (LC3B; also known as MAP1LC3B), an important component of the canonical autophagy machinery (Huang and Brumell, 2014; Klionsky et al., 2021; Thurston et al., 2009; Wild et al., 2011; Zheng et al., 2009). In the case of HeLa cell infection, *S. flexneri* septin cages are known to colocalize with ubiquitin (Mostowy et al., 2010, 2011). New work performed *in vitro* using purified proteins has shown that septins can directly bind the outer membrane of growing *S. flexneri* cells (Lobato-Márquez et al., 2021). However, whether septins or bacterial membrane components are ubiquitylated, and the type of ubiquitin linkage employed by the host-cell during cage entrapment was unknown. Although the bacterial septin cage is considered a paradigm for the investigation of cytoskeleton–autophagy interactions (Mostowy and Shenoy, 2015; Van Ngo and Mostowy, 2019; Welch and Way, 2013), we still lack fundamental understanding of how septin-cage-entrapped bacterial cells are targeted to autophagy.

Recent advances in microscopy have revolutionized cellular microbiology and the study of host–pathogen interactions (López-Jiménez and Mostowy, 2021). Cryo-soft X-ray tomography (cryo-SXT) is a technique that permits imaging of unstained and cryopreserved biological samples (in their near-native state) as thick as 10 µm, therefore overcoming most electron microscopy (EM) limitations (Carrascosa et al., 2009; Conesa et al., 2016; Harkiolaki et al., 2018; Schneider et al., 2010). In addition, cryo-SXT possesses a resolving power in the nanometer scale (as high as 30 nm) and high contrast for biological samples, making cryo-SXT an ideal technique to study the precise localization and organization of membrane-based organelles and pathogens within the host cell cytosol (Carrascosa et al., 2009; Conesa et al., 2020; Cruz-Adalia et al., 2014; Groen et al., 2019; Kounatidis et al., 2020). In this report, we use correlative light and cryo-SXT, Airyscan confocal microscopy and live-cell imaging to study septin–autophagy interactions during *S. flexneri* cage entrapment.

## RESULTS

### Visualization of mitochondrial morphology during *S. flexneri* infection by correlative light and cryo-SXT

**Fig. 1.**
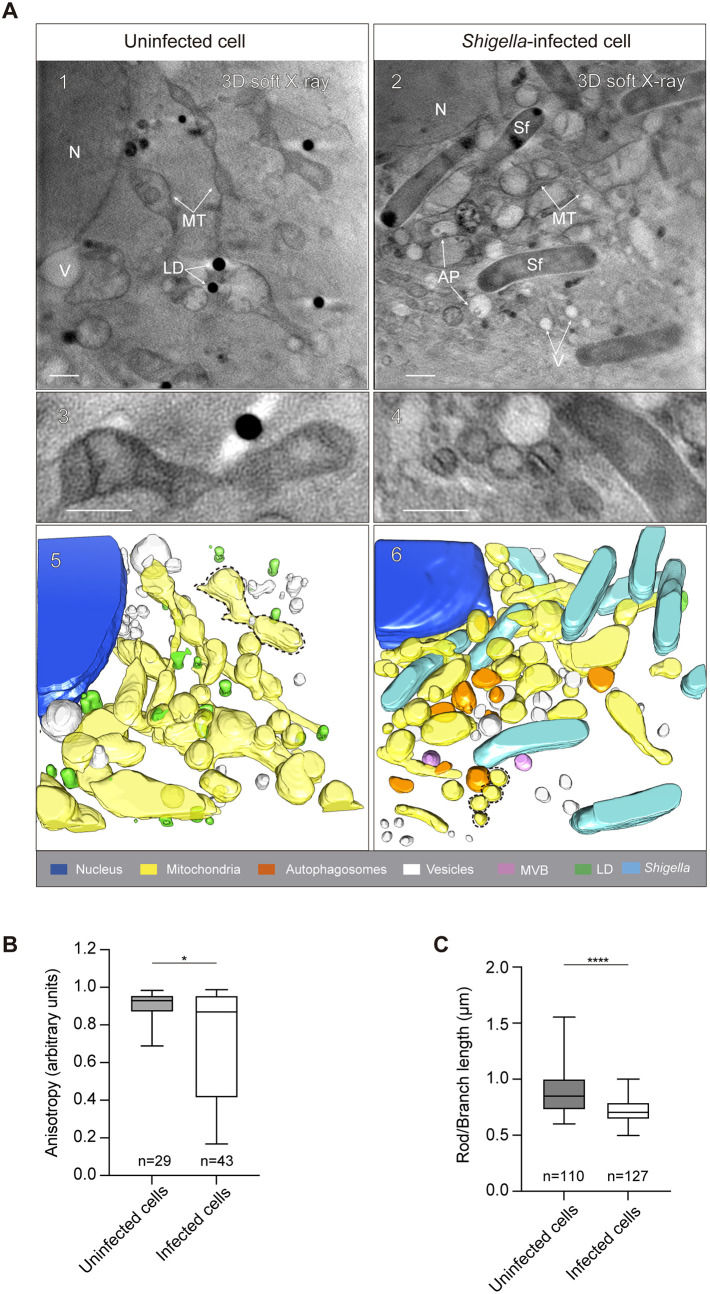
**Visualization of mitochondrial dynamics during *Shigella flexneri* infection by correlative fluorescence and cryo-SXT.** (A) Uninfected (left panels) and *S. flexneri*-infected (right panels) HeLa cells where stained with Mitotracker Red and imaged by correlative light and cryo-SXT. (1, 2) Tomographic slices showing the elongated mitochondrial network in uninfected cells (left panel), and fragmented mitochondria during *S. flexneri* infection (right panel). Images shown corresponds to slices of 3.9 µm (1) and 4.1 µm thickness. AP, autophagosome; LD, lipid droplet; MT, mitochondria; N, nucleus; V, vesicles. (3, 4) Magnification of mitochondria from 1 and 2. (5, 6) Volumetric representation of the tomograms in 1 and 2. Scale bars: 1 μm. (B) Quantification of mitochondrial elongation using cryo-SXT. Data represent anisotropy from *n*=29 mitochondria (uninfected cells) from two independent tomograms and *n*=43 mitochondria (*S. flexneri*-infected cells) from three independent tomograms. *P=0.038 (Mann–Whitney test). (C) Quantification of mitochondrial branch length (a measurement of mitochondrial network elongation) using epifluorescence microscopy and Fiji plugin MiNa. Data show mitochondrial rod/branch length from *n*=109 mitochondria (uninfected cells) and *n*=127 mitochondria (*S. flexneri*-infected cells) distributed in two independent experiments. In B, C, the box represents the 25–75th percentiles, and the median is indicated. The whiskers show the range. ****P<0.0001 (Mann–Whitney test).

We applied a correlative light and cryo-SXT pipeline to *S. flexneri*-infected HeLa cells (Figs S1 and S2). Given that *S. flexneri* is reported to fragment mitochondria during host cell invasion (Carneiro et al., 2009; Sirianni et al., 2016), we first studied mitochondrial morphology during bacterial infection. We stained HeLa cells with Mitotracker Red and infected them (or not) with *S. flexneri* for 3 h. In the absence of infection, Airyscan confocal microscopy showed that HeLa cells possessed elongated mitochondria (Fig. S3, top panel) and cryo-SXT demonstrated elongated mitochondria forming an intricate network (Fig. 1A, left panels). By contrast, infection with *S. flexneri* caused mitochondrial fragmentation (Fig. 1A right panels; Fig. S3, bottom panel); in this case mitochondria were dramatically smaller and less interconnected as compared to those in uninfected cells. We quantified the length of mitochondria using both fluorescence microscopy and cryo-SXT. Consistent with our previous report that *S. flexneri* fragments mitochondria during host cell invasion (Sirianni et al., 2016), *S. flexneri*-infected HeLa cells showed significantly shorter mitochondria as compared to uninfected HeLa cells (Fig. 1B,C). Of note, in the absence of fluorescence markers labelling cytosolic bacteria, the identification of *S. flexneri* was obvious by cryo-SXT (Fig. 1 and Fig. S2). Together, these data confirm that *S. flexneri* fragments mitochondria during infection and highlights the potential of cryo-SXT to study other intracellular hallmarks of the *S. flexneri* infection process.

### Use of cryo-SXT to study *S. flexneri* entrapment in septin cages *in situ*

**Fig. 2.**
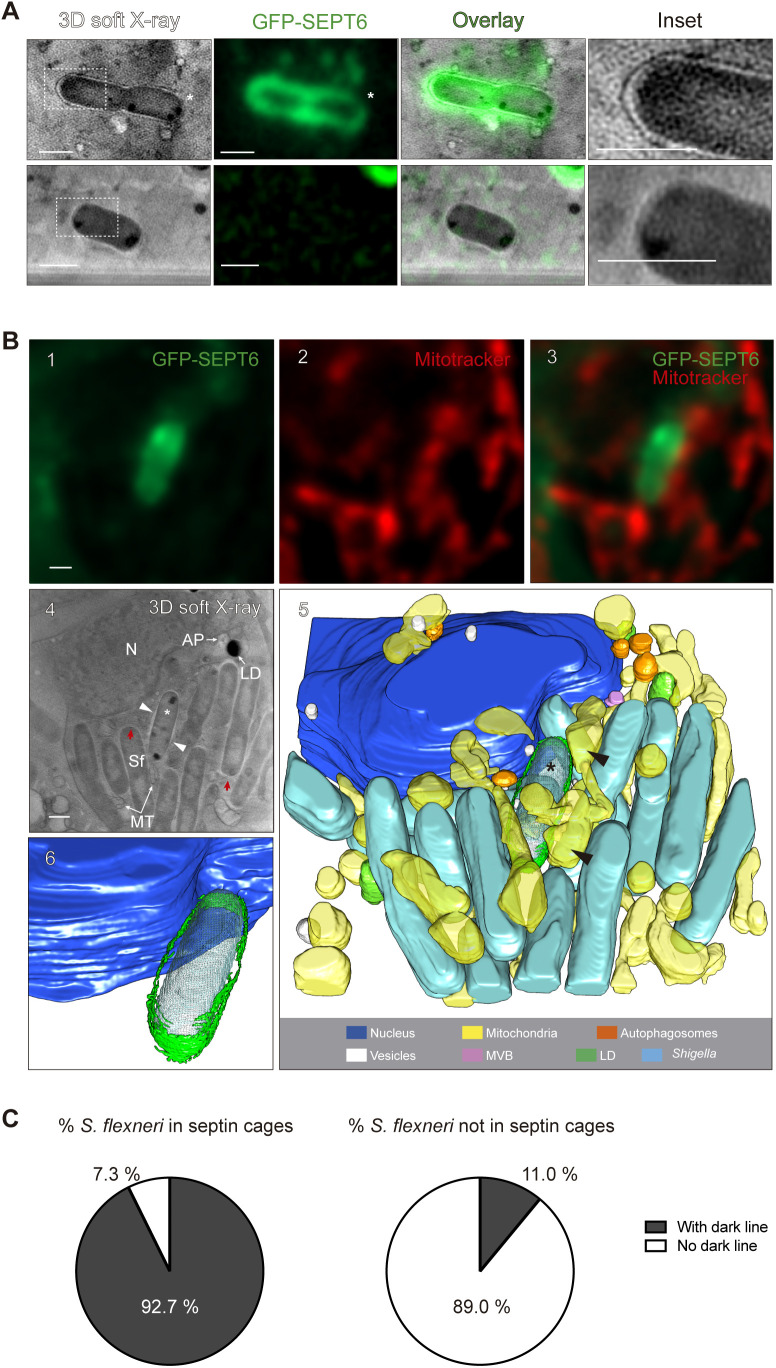
**Fluorescent signal of septin cages correlates to increased soft X-ray densities.** (A) Representative example (from *n*=51 bacteria) of a bacterium entrapped in a septin cage imaged by correlative light and cryo-SXT. Soft X-ray dense lines correlate with GFP–SEPT6 fluorescence (top panel inset). Note that where the septin signal is weaker, the X-ray signal is also less intense (white asterisk, top panel). Representative example (from *n*=97 bacteria) of a bacterium not entrapped in a septin cage imaged by correlative light and cryo-SXT. Soft X-ray dense lines are not observed around non-caged bacteria (bottom panel). See also Fig. S4. Scale bars: 1 μm. (B) Representative cellular environment from *n*=23 tomograms of a septin-caged bacterium. (1–3) Fluorescence microscopy of septin-caged bacterium. (4) Tomographic slice of the same area imaged on 1–3. Image shown corresponds to a slice of 4.6 µm thickness. See also Movie 1. AP, autophagosome; N, nucleus; LD, lipid droplets; MT, mitochondria; Sf, *S. flexneri*; red arrows point to extended bacterial periplasm at the pole of some cells; white arrowheads point to the dense X-ray structure surrounding septin-caged *S. flexneri*; *septin-caged bacterium. Note elongated mitochondria surrounding the septin-cage-entrapped bacterium. (5) Volumetric representation of the tomogram on panel 4. (6) Image of the the 3D architecture of the *S. flexneri* septin cage, highlighting septins surrounding the entrapped bacterium. Scale bars: 1 μm. (C) Percentage of *S. flexneri* entrapped or not in septin cages (as defined by fluorescence microscopy) that show a dense X-ray structure surrounding the bacterial cell. Data represent *n*=55 (septin-caged *S. flexneri* of which 51 show dense X-ray structure) and *n*=109 (non-caged *S. flexneri* of which only 12 show dense X-ray structure) bacteria distributed in 53 tomograms.

The *S. flexneri* septin cage has been studied for over 10 years using tissue culture cells, zebrafish infection models and a wide variety of fluorescent microscopy techniques (Krokowski et al., 2018; Mostowy et al., 2010, 2013, 2011; Sirianni et al., 2016). More recently, we imaged bacterial septin cages reconstituted *in vitro* (using purified proteins) at the nanometer scale using cryo-electron tomography, and in this case resolved how septins interact with bacterial membrane (Lobato-Márquez et al., 2021). Despite these efforts, septin cages have not been imaged at high-resolution in their native state during host cell infection. To address this, we employed correlative light and cryo-SXT to visualize septin cage entrapment of *S. flexneri in situ*. HeLa cells producing GFP–SEPT6 (Sirianni et al., 2016) were infected for 3 h with *S. flexneri*, plunge-freezed and introduced into our correlative pipeline (Fig. S1). Bacterial septin cages identified by epifluorescence microscopy were subsequently imaged by X-ray tomography. Strikingly, 92.7% of *S. flexneri* cells entrapped in septin cages could be identified by cryo-SXT as an X-ray-dense structure (Fig. 2A, top panel; Fig. S4A). In these images, GFP–SEPT6 fluorescence colocalizes with the X-ray-dense structure, and when GFP–SEPT6 fluorescence is absent the X-ray-dense signal is also absent, strongly suggesting that dark features surrounding bacterial membrane correspond to structures (probably containing host lipids) that are enriched in septins (Fig. 2A, top panel; Fig. S4A). Consistent with this, the use of correlative light and cryo-SXT revealed that only 11% of bacteria not clearly entrapped in GFP–SEPT6 septin cages show an X-ray-dense structure (Fig. 2A, bottom panel, Fig. 2C).

In contrast to the dense X-ray structures surrounding *S. flexneri* colocalizing with the GFP–SEPT6 fluorescence signal of the septin cage, we occasionally observed a thin X-ray signal at the pole of some bacterial cells (Figs 2B, panel 4, red arrows). We hypothesized that this thin X-ray signal, which does not colocalize with the GFP–SEPT6 signal, might correspond to the outer membrane of bacteria that exhibit an extended periplasm. To test this, we combined our *in vitro* septin cage reconstitution assay using purified septin complexes (SEPT2–GFP-SEPT6–SEPT7) (Lobato-Márquez et al., 2021) with correlative light and cryo-SXT. As expected, recombinant septin proteins did not provide enough density to be visualized by cryo-SXT (Fig. S4B), reinforcing our conclusion that the X-ray-dense structures surrounding bacterial cells are enriched in host cell-derived lipids. In support of our hypothesis that thin X-ray signal present at some bacterial cell poles represents bacterial outer membrane (with an extended periplasm), we could visualize the *S. flexneri* outer membrane by cryo-SXT using bacteria grown in broth (Fig. S4B). Consistent with this, previous work has shown that the periplasm of Gram-negative bacteria expands at the bacterial cell poles under stressful conditions (such as bacteria grown in minimal medium or high osmolarity) (Sochacki et al., 2011).

Mitochondria promote *S. flexneri* septin cage assembly (Sirianni et al., 2016). In agreement with this, epifluorescence microscopy showed that septin cages are surrounded by elongated mitochondria (Fig. 2B, panels 1–3; Movie 1), and cryo-SXT revealed that those septin-caged bacteria are tightly associated with an elongated mitochondrial network (Fig. 2B, panels 4 and 5; Movie 1). These observations are in sharp contrast with the cellular environment of *S. flexneri* not entrapped by septin cages (Fig. 1), where mitochondria are clearly more fragmented and the mitochondrial network less extensive. Together, cryo-SXT enables the *in situ* visualization of *S. flexneri* septin cages as X-ray-dense structures in close contact with elongated host cell mitochondria.

### Interaction of septins with LC3B-positive membranes during *S. flexneri* entrapment

**Fig. 3.**
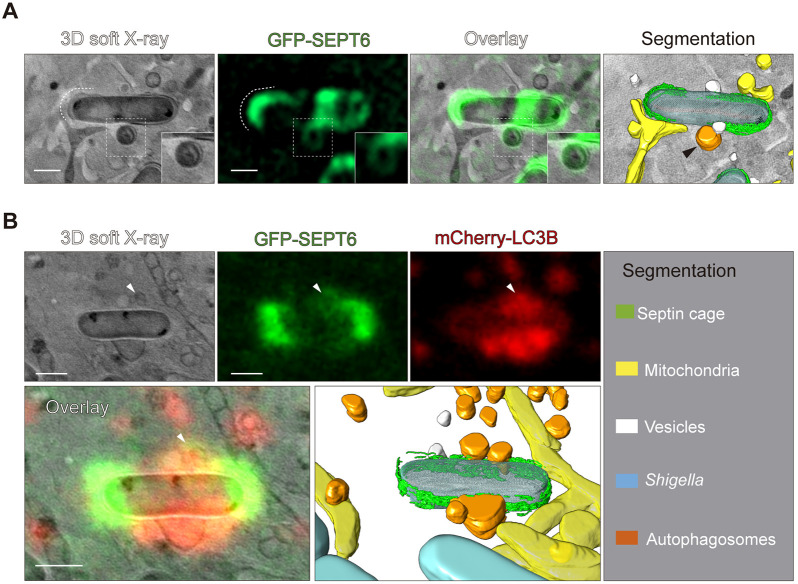
**Septins interact with LC3B-decorated vesicles during autophagy of *S. flexneri*.** (A) Correlative light and cryo-SXT showing that septins localize to vesicles interacting with septin-caged *S. flexneri* (black arrow). Scale bar: 1 μm. (B) Correlative light and cryo-SXT showing septins localize to LC3B-decorated vesicles (arrowheads) recruited to *S. flexneri*. Images shown correspond to a slice of 2.6 µm thickness. See also Movie 2. Scale bars: 1 μm. Images in A and B represent two examples extracted from 14 tomograms.

How septins interact with the autophagy machinery to clear *S. flexneri* is unknown. During visualization of *S. flexneri*–septin cages by correlative light and cryo-SXT we observed septin–autophagosome interactions (Fig. 3A). To confirm that the vesicles contacting caged bacterial cells are autophagosomes, we infected HeLa cells stably producing GFP–SEPT6 and transfected with mCherry–LC3B with *S. flexneri* and performed correlative light and cryo-SXT. We observed LC3B-positive vesicles recruited to septin-caged bacteria, supporting the hypothesis that septins and autophagic membranes interact during autophagy of entrapped *S. flexneri* (Fig. 3B; Movie 2). In all cases, *S. flexneri* septin cages were tightly bound to host cell mitochondria (Fig. 3A,B). To visualize septin–autophagosome interaction in real time, we performed time-lapse epifluorescence microscopy using HeLa cells stably producing GFP–SEPT6 and transfected with mCherry–LC3B. Consistent with the model that septin cage entrapment and autophagy are interdependent processes (Mostowy et al., 2010, 2011), live-cell imaging showed a coordinated recruitment of GFP–SEPT6 and mCherry–LC3B to cytosolic *S. flexneri* (Fig. S5, Movies 3 and 4).

To study the dynamics of septin–LC3B interactions at high resolution, we performed time-lapse Airyscan confocal microscopy of HeLa cells stably producing GFP–SEPT6 and transfected with mCherry–LC3B infected with *S. flexneri* (Fig. S6A). Live-cell imaging revealed that septins interact with LC3B-positive membranes during autophagy of septin-caged bacterial cells (Fig. S6A, white arrowheads, Movie 5). In this case, septins assemble as ∼0.6 µm ring-like structures surrounding LC3B-positive membranes (Fig. S6A,B, Movie 6), consistent with septin–Atg8 interactions during autophagosome formation in *Saccharomyces cerevisiae* (Barve et al., 2018). Recent work using different cell types has shown a role for septins during autophagosome formation that depends on direct septin–LC3B interactions (Barve et al., 2018; Toth et al., 2022). Considering this, we hypothesized that septins might also bind to LC3B during autophagy of caged *S. flexneri*. To test this, we performed co-immunoprecipitation assays using GFP–LC3B-producing HeLa cells; however, under the conditions tested we could not detect septin–LC3B binding (data not shown). Together, cryo-SXT and live-cell imaging data suggest an interaction between septins (which are well known as membrane-interacting proteins) and autophagic membranes during autophagy of entrapped *S. flexneri* cells.

### K63-linked ubiquitin chains decorate septin-cage-entrapped *S. flexneri* promoting their targeting for autophagy

**Fig. 4.**
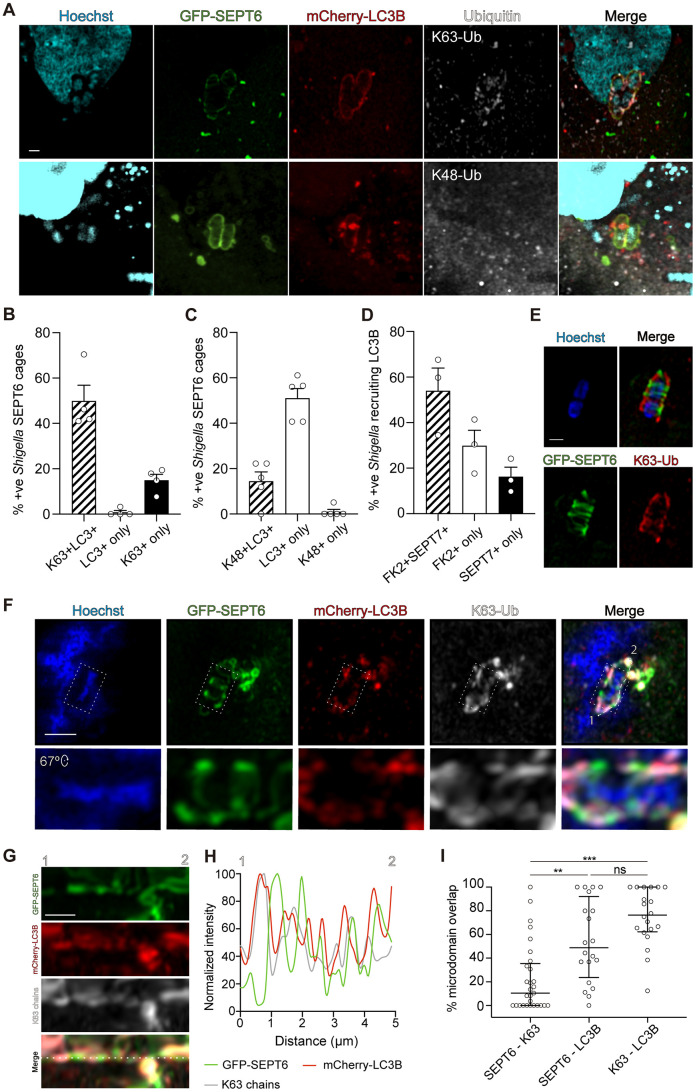
**K63-linked ubiquitin chains target *S. flexneri* to autophagy.** (A) Airyscan confocal image showing septin-caged *S. flexneri* colocalizing with LC3B and K63 polyubiquitin (top panel) and septin caged *S. flexneri* colocalizing with LC3B but not K48 polyubiquitin (bottom panel). Scale bar: 1 μm. (B) Quantification of *S. flexneri* septin cages colocalizing with LC3B and/or K63 polyubiquitin. Data represent the mean±s.e.m. from *n*=66 *S. flexneri* with septin cages distributed in four independent experiments. (C) Quantification of *S. flexneri* with septin cages colocalizing with K48 chains. Data represents the mean±s.e.m. from *n*=191 *S. flexneri* septin cages distributed in five independent experiments. (D) Quantification of *S. flexneri* decorated with GFP–LC3B colocalizing with ubiquitin (FK2) and/or SEPT7. Data represent the mean±s.e.m. from *n*=149 bacteria distributed in three independent experiments. (E) Airyscan confocal images showing the formation of separate microdomains of GFP-SEPT6 and K63 polyubiquitin on the surface of *S. flexneri*. Scale bar: 1 μm. (F) Airyscan confocal images showing the formation of separate microdomains of septins, K63 polyubiquitin and LC3B on the surface of *S. flexneri*. mCherry–LC3B partially colocalized with both GFP–SEPT6 and K63 chains. Scale bar: 2 μm. (G) Representation of the GFP–SEPT6, K63 polyubiquitin and mCherry-LC3B microdomains of the septin cage from E. 1 and 2 mark the beginning and end of the dashed white line from F. Scale bar: 1 µm. (H) Fluorescence intensity profiles of GFP–SEPT6, K63 polyubiquitin and mCherry-LC3B across the dashed white line shown in the bottom panel of G. (I) Quantification of the colocalization between GFP-SEPT6, K63 polyubiquitin and mCherry–LC3B. Data represent the median±interquartile range from *n*=29 (GFP-SEPT6 and K63 polyubiquitin), *n*=20 (K63 chains and mCherry-LC3B) and *n*=20 (GFP-SEPT6 and mCherry-LC3B) bacteria distributed in three independent experiments. *P<0.001; ***P<0.0001; ns, non-significant (Kruskal–Wallis with Dunn's post-test).

How septin-cage-entrapped *S. flexneri* are recognized by autophagy machinery is poorly understood (Mostowy et al., 2010, 2011). K63 and K48 polyubiquitin chains are responsible for targeting cargoes to autophagic or proteasomal degradation, respectively (Akutsu et al., 2016). During xenophagy, ubiquitin is recognized by autophagy adaptor proteins that also bind LC3B. To further explore how septin-caged *S. flexneri* are targeted to autophagy, we tested whether entrapped bacteria are modified by K63 or K48 polyubiquitin chains. We infected HeLa cells producing mCherry–LC3B with *S. flexneri* and quantified the percentage of septin-caged bacteria colocalizing with K63 or K48 polyubiquitin (Fig. 4A). Consistent with a role for K63 chains (and not K48 chains) in xenophagy, 49.9±6.9% (mean±s.e.m.) of septin-cage-entrapped *S. flexneri* colocalized with both K63 polyubiquitin and LC3B (Fig. 4B), whereas only 14.5±4.1% of caged bacteria colocalized with both K48 polyubiquitin and LC3B (Fig. 4C). In support of a role for K63 in promoting the recruitment of LC3B, only 0.8±0.8% of septin-caged *S. flexneri* colocalized with LC3B but not K63 polyubiquitin (Fig. 4B). In agreement with a role for septin cages in targeting bacteria to xenophagy, infection of GFP–LC3B-producing HeLa cells showed that 54±10% of GFP–LC3B-positive *S. flexneri* also colocalized with ubiquitin and septins (Fig. 4D).

In addition to septin cage entrapment, the host cell can restrict actin-based motility of *S. flexneri* by decorating cytosolic bacteria with guanylate-binding proteins (GBPs) (Li et al., 2017; Piro et al., 2017; Wandel et al., 2017). However, *S. flexneri* can escape from GBP recognition by secreting an E3-ubiquitin ligase (IpaH9.8) that targets GBPs with K48 polyubiquitin for proteasomal degradation (Li et al., 2017; Wandel et al., 2017). Consistent with this, the mutant *S. flexneri* Δ*ipaH9.8* cannot ubiquitylate GBPs and is recognized by GBPs more frequently than wild-type (WT) bacteria (Li et al., 2017; Wandel et al., 2017). Considering this, we questioned whether *S. flexneri* can target septins for proteasomal degradation in order to escape from cage entrapment. To test this, we infected HeLa cells with *S. flexneri* WT or the E3 ligase mutants Δ*ipaH1.4*, Δ*ipaH2.5* or Δ*mxiE* (a strain lacking a transcription factor responsible for the upregulation of many effector genes, including 12 *S. flexneri*-encoded E3 ligases) and quantified the percentage of SEPT7 cages. From these experiments, we did not observe significant differences between *S. flexneri* WT (11.5±0.8%), Δ*ipaH1.4* (12.5±0.9%), Δ*ipaH2.5* (10.7±1.4%) and Δ*mxiE* (12.8±0.9%) (mean±s.e.m.), indicating that the E3 ligases tested here do not enable escape from septin cage entrapment (Fig. S7). We do not rule out the possibility of mechanistic redundancy among *S. flexneri* E3 ligases, yet these observations are in agreement with previous work showing that proteasome inhibition does not alter the percentage of septin-caged *S. flexneri* (Mostowy et al., 2010).

### K63-linked ubiquitin chains and septins are present in non-overlapping microdomains around *S. flexneri*

To test whether septins can promote the ubiquitylation of cytosolic *S. flexneri*, we depleted SEPT7 with small interfering RNA (siRNA) (Figs S8A,B) and quantified the percentage of total cytosolic bacteria decorated with total ubiquitin (FK2). Analysis by confocal microscopy revealed no significant difference in the percentage of ubiquitin-positive *S. flexneri* between control (6.2±0.9%) and SEPT7 (5.8±0.4%) (mean±s.e.m.) siRNA-treated cells (Figs S8C,D). We observed a similar recruitment of K63 polyubiquitin to *S. flexneri* in the case of control (6.2±1.2%) and SEPT7 (5.8±1.2%) siRNA-treated cells (Fig. S8E). These data suggest that recruitment of K63 chains to *S. flexneri* is independent from the recruitment of SEPT7. To test this, we visualized ubiquitylated *S. flexneri* (K63 chains) entrapped in septin cages during the infection of HeLa cells stably producing GFP–SEPT6 and transfected with mCherry–LC3B. Strikingly, Airyscan confocal microscopy showed that GFP–SEPT6 and K63 polyubiquitin are present in non-overlapping microdomains around the bacterial membrane (Fig. 4E, Fig. S9). We then visualized the distribution of GFP–SEPT6, K63 chains and mCherry–LC3B on cytosolic *S. flexneri*. Whereas SEPT6 and K63 polyubiquitin were present in different microdomains on the bacterial membrane, LC3B partially colocalized with both SEPT6 and K63 polyubiquitin (Fig. 4F,G,H). We quantified the percentage of GFP-SEPT6, K63 polyubiquitin and mCherry-LC3B colocalizing with each other. In agreement with septins and K63 polyubiquitin recognizing separate *S. flexneri* microdomains, SEPT6 rarely (median 10.5%) colocalized with K63 chains (Fig. 4I). Consistent with their role in targeting substrates to autophagy, K63 polyubiquitin frequently (median 76.4%) colocalized with LC3B. Surprisingly, LC3B colocalized with both SEPT6 and K63 polyubiquitin equally (Fig. 4I). Together, these data demonstrate that SEPT6 and K63 polyubiquitin are present in separate bacterial microdomains during autophagy of septin-cage-entrapped *S. flexneri*.

## DISCUSSION

The septin cage was first described over 10 years ago (Mostowy et al., 2010; Robertin and Mostowy, 2020). In the case of *S. flexneri*, septins have been shown to bind bacterial membrane for cage entrapment *in vitro* (Lobato-Márquez et al., 2021). Despite intensive research, septin cages had not yet been imaged in their native state inside infected cells, and the interplay between septins and autophagy was unknown. Here, we used correlative light and cryo-SXT to visualize septin-cage-entrapped bacterial cells at the nanometer scale to understand their link with autophagosome formation. We discovered that septin cages are strongly correlated with X-ray-dense structures surrounding *S. flexneri*. In agreement with a role for mitochondria during cage entrapment of *S. flexneri*, cryo-SXT showed that mitochondria are tightly bound to septin cages *in situ*. We also showed that septin-caged *S. flexneri* are decorated with K63 polyubiquitin (via a process separate from recruitment of septins), which targets entrapped bacteria for autophagy. Airyscan confocal microscopy and correlative light and cryo-SXT suggest that septins interact with LC3B-decorated membranes during autophagy of *S. flexneri*. Taken together, a model emerges, where: (1) septins recognize poles of cytosolic *S. flexneri* for cage entrapment; (2) unknown E3-ubiquitin ligase(s) decorate separate regions of the bacterial membrane (not covered by septins) with K63 polyubiquitin; (3) autophagy adaptor proteins link ubiquitylated bacteria to LC3B; and (4) septins interact with LC3B-positive membranes during autophagy of *S. flexneri*. This process ultimately leads to the encapsulation of septin-cage-entrapped *S. flexneri* into autophagosomes targeted to lysosomal fusion.

Our data show that the host cell ubiquitin machinery targets microdomains on the bacterial membrane that are distinct from the microdomains bound to septins. Ubiquitylation is a sequential enzymatic cascade mediated by ubiquitin-activating (E1), ubiquitin-conjugating (E2), and ubiquitin-ligating (E3) enzymes (Hershko and Ciechanover, 1998). In the human genome, there are two E1 genes, 30–50 E2 genes and >600 E3-encoding genes (Zheng and Shabek, 2017); this diversity has made it highly challenging to identify the specific enzyme(s) targeting *S. flexneri*. The E3-ubiquitin ligase LRSAM1 has been shown to decorate a *S. flexneri* Δ*icsB* mutant (IcsB is an effector correlated with autophagy and septin cage avoidance) with K63 and K27 polyubiquitin (Huett et al., 2012; Mostowy et al., 2010). For this study, we tried to ubiquitylate *S. flexneri in vitro* using purified E1, UBE2D2 and LRSAM1, but under the conditions tested we could not detect ubiquitylated *S. flexneri* (data not shown). As far as we know, the E3-ubiquitin ligase(s) that decorate *S. flexneri* WT with K63 polyubiquitin have not yet been identified. The E3 ligase RNF213 has recently been shown to ubiquitylate Lipid A on the outer membrane of *S*. Typhimurium (Otten et al., 2021). Considering the structure of Lipid A is conserved between *S. flexneri* and *S*. Typhimurium, it is tempting to speculate that RNF213 also ubiquitylates *S. flexneri*. As both septins (Lobato-Márquez et al., 2021) and RNF213 (Otten et al., 2021) target the bacterial membrane, this could explain the distinct microdomains we observed during *S. flexneri* infection. New technologies in cellular microbiology (López-Jiménez and Mostowy, 2021), such as proximity-dependent biotinylation coupled with mass spectrometry (Liu et al., 2020), might prove useful in identifying the specific E3-ubiquitin ligase(s) targeting *S. flexneri*. Alternatively, high-throughput microscopy combined with CRISPR-Cas libraries has been used to identify the E3-ubiquitin ligases involved in *S.* Typhimurium ubiquitylation (Heath et al., 2016; Polajnar et al., 2017). Posttranslational modifications are well known to play an important role in host–pathogen interactions (Chambers and Scheck, 2020). Septins can be post-translationally modified by ubiquitylation, SUMOylation, acetylation and phosphorylation (Hernandez-Rodriguez and Momany, 2012). We recently demonstrated that purified septin proteins can recognize bacterial membranes in the absence of additional host cell factors, including posttranslational modifications (Lobato-Márquez et al., 2021). Together with data showing that K63 polyubiquitin does not colocalize with septins at the *S. flexneri* septin cage, we propose that K63 polyubiquitin is not required for septin cage assembly. Considering that SUMOylation has been shown to regulate septin assembly during cytokinesis (Hernandez-Rodriguez and Momany, 2012; Ribet et al., 2017), it is next of great interest to study the role of posttranslational modifications (including SUMO and ubiquitin linkages other than K63 polyubiquitin) during septin-mediated cell-autonomous immunity.

Cryo-SXT is a powerful imaging technique used to study membrane-based cellular organelles, including autophagy. Here, we used correlative light and cryo-SXT to study septin–autophagy interactions during *S. flexneri* cage entrapment. During autophagy of *S. flexneri*, we show that septins interact with LC3B-positive membrane. Unfortunately, the resolution obtained in our study using cryo-SXT did not permit the visualization of individual septin filaments, nor how they interact with autophagic membranes, and thus the precise role of septin–LC3B interactions during autophagy of *S. flexneri* requires further investigation. Considering the recent visualization of septin filaments on septin cages reconstituted *in vitro* (Lobato-Márquez et al., 2021), and the combination of focused ion beam milling with cryo-electron tomography (Wagner et al., 2020), it will be important to explore *in situ* how septins organize on autophagic membrane at nanometer resolution. In parallel, combining purified septin proteins with autophagosomes reconstituted *in vitro* might illuminate the molecular mechanisms underlying septin–autophagosome interactions (Chang et al., 2021; Sawa-Makarska et al., 2020). In this way, an in depth understanding of septin–autophagy interactions could help to identify novel approaches for bacterial infection control.

## MATERIALS AND METHODS

### Reagents

The following antibodies were used: rabbit anti-SEPT7 (#18991, IBL), rabbit anti-ubiquitin Lys-63-specific (#05-1308, Merck), rabbit anti-ubiquitin Lys-48-specific (#05-1307, Merck), mouse FK2 (#PW8810, Enzo Life Sciences), Alexa-555-conjugated anti-rabbit antibody (#10082602, Thermo Fisher Scientific), Alexa-647-conjugated anti-rabbit antibody (#A27040, Thermo Fisher Scientific). Hoechst 33342 (#H3570, Thermo Fisher Scientific) was used through the manuscript to stain for *S. flexneri* and host cell DNA.

### Bacterial strains and culture conditions

Unless otherwise indicated, *Shigella flexneri* 5a str. M90T producing the adhesin AfaI (Mostowy et al., 2010) was used throughout the manuscript. *S. flexneri* was grown in trypticase soy broth (TCS)-agar containing 0.01% (w/v) Congo Red (Sigma-Aldrich; #C6767) to select for red colonies, indicative of a functional type three secretion system (T3SS). Conical polypropylene tubes (#CLS430828, Corning) containing 5 ml of TCS were inoculated with individual red colonies of *S. flexneri* and were grown ∼16 h at 37°C with shaking at 200 rpm. The following day, bacterial cultures were diluted in fresh pre-warmed TCS (1:50 v/v), and cultured until an optical density (OD, measured at 600 nm) of 0.6 was reached. To maintain the plasmid encoding *afaE* (AfaI-encoding gene), TCS was supplemented with 100 µg/ml of carbenicillin (Sigma-Aldrich; #C1389).

*Escherichia coli* strains were grown in lysogeny broth (LB; Invitrogen) in conical polypropylene tubes at 37°C with shaking at 220 rpm. *E. coli* DH5α was used to purify pKD46 and pKD4 plasmids (Datsenko and Wanner, 2000), and LB was supplemented with 100 μg/ml of carbenicillin or 50 μg/ml of kanamycin, respectively. *E. coli* was also used to purify the plasmid encoding mCherry–LC3B; in this case, this strain was grown in LB supplemented with 100 μg/ml of carbenicillin. Bacterial stocks were stored in 10% glycerol at −80°C.

### Design of bacterial mutant strains

Primers used in this study were designed using Benchling (https://benchling.com) and are listed in Table S1. *S. flexneri* mutants were engineered using λ-Red-mediated recombination (Datsenko and Wanner, 2000). In brief, kanamycin resistance-encoding DNA cassettes were amplified using pKD4 plasmid as template and primers containing 50 bp nucleotides homologous to the site of insertion. Resulting DNA fragments were electroporated in *S. flexneri* electrocompetent cells producing λ-Red recombinase and plated in TSA plates supplemented with 0.01% of Congo Red and 50 μg/ml of kanamycin. All strains were verified by PCR.

### Mammalian cell culture

HeLa (ATCC CCL-2) cells were grown at 37°C and 5% CO_2_ in Dulbecco's Modified Eagle Medium (DMEM, GIBCO) supplemented with 10% fetal bovine serum (FBS, Sigma-Aldrich). GFP–SEPT6-producing HeLa cells (Sirianni et al., 2016) were grown as above in DMEM supplemented with 10% FBS and 2 µg/ml of puromycin (Merck; #P8833). GFP–LC3B-producing HeLa cells (Runwal et al., 2019) were grown as above in DMEM supplemented with 10% FBS.

### HeLa cell plasmid transfection

8×10^4^ HeLa cells were seeded in 6-well plates (Thermo Fisher Scientific) containing 22×22 mm glass coverslips, Quantifoil (R2/2, Quantifoil Micro Tools) Au-EM finder grids, coated with holey carbon, or MatTek dishes 2 days before transfection. Plasmid transfections were performed in 1 ml DMEM with 250 ng of a plasmid encoding *mCherry-LC3B* (a gift from the Sharon Tooze laboratory, Molecular Cell Biology of Autophagy Laboratory, The Francis Crick Institute, UK) using JetPEI (Polyplus transfection) as described in Mazon Moya et al. (2014) and incubated at 37°C and 5% CO_2_ for 6 h. Then, the medium was replaced with 2 ml of fresh pre-warmed DMEM supplemented with 10% FBS until the following day when cells were infected.

### Infection of human cells

For experiments involving paraformaldehyde (PFA)-fixed samples (Figs 1C, 4; Figs S3, S7, S8 and S9) 9×10^4^ HeLa cells were seeded in 6-well plates (Thermo Fisher Scientific) containing 22×22 mm glass coverslips 2 days before the infection. For experiments involving live-cell imaging (Figs S5 and S6), 9×10^4^ HeLa cells were seeded in MatTek glass-bottom dishes (MatTek corporation). For experiments involving correlative light and cryo-SXT (Figs 1A, 1B, 2, 3; Figs S1 and S4), 8×10^4^ HeLa cells were seeded in 6-well plates containing Quantifoil Au-EM finder grids. Bacterial cultures were grown as described above, and cell cultures were infected with *S. flexneri afaE* at a multiplicity of infection (MOI, bacteria:cell) of 10:1. Then, plates were placed at 37°C and 5% CO_2_ for 30 min. Infected cultures were washed 2× with phosphate-buffered saline (PBS) pH 7.4 and incubated with fresh DMEM containing 10% FBS and 50 mg/ml gentamicin (Merck; #G1264) at 37°C and 5% CO_2_ up to 3 h. For live-cell imaging experiments, DMEM was replaced by OPTI-MEM containing 10% FBS and 50 mg/ml gentamicin and MatTek dishes placed on an Airyscan 880 confocal microscope coupled to a temperature-controlled incubator (37°C).

### Cryo-epifluorescence microscopy

After infection, HeLa cells were fixed by plunge-freezing using a Vitrobot Mark IV (Thermo Fisher Scientific). Prior to vitrification, samples were slightly fixed with 1% PFA for 5 min to inactivate bacteria and imaged using an Axiovert Z1 driven by ZEN Blue 2.3 software (Carl Zeiss) epi-fluorescence microscope or a confocal microscope LSM710 (Carl Zeiss) driven by ZEN 2010 software. To stain for mitochondria, samples were incubated with 100 nM of Mitotracker Red (Invitrogen) for 30 min before vitrification. In all cases, grids were incubated with 100 nm fiducial gold nanoparticles (that would help during the alignment of the tilt series) for 30 s before vitrification. Following vitrification, grids were shipped to the Mistral beamline at the ALBA synchrotron (Barcelona, Spain). Vitrified grids were then transferred in liquid nitrogen to the cryo-correlative cooling stage Linkam CSM196 (Linkam Scientific Instruments) to hold samples at a stable −190°C during analysis. The cryo-stage was inserted into an AxioScope A1 (Carl Zeiss) epifluorescence microscope with a N-Achroplan 50×/0.6 Ph1 objective and imaged with a CCD AxioCam ICm1 (Carl Zeiss).

Cryo-fluorescence microscopy was used to pre-select vitrified samples and map the position of cells. Selected samples were then transferred to the Mistral synchrotron beamline at liquid nitrogen temperature.

### Soft X-ray cryo-tomography

Grids were visualized on-line with a visible light microscope integrated within the X-ray microscope to correlate cell positions identified with epifluorescence and cryo-epifluorescence images prior to sample loading into the Mistral beamline. Zero-degree soft X-ray projection mosaics were acquired to image the areas of interest and define the tomogram acquisition areas. Tilted-series were acquired at 520 eV photon energy from −70° to 70° for each degree, using a 25-nm zone plate. The exposure time used was associated to sample conditions (thickness) and ranged from 1 to 4 s. Pixel size was set to 11.5 nm.

Tilted series were normalized to the flatfield using the XMIPP 3 software package (de la Rosa-Trevin et al., 2013), aligned with IMOD (Kremer et al., 1996) and reconstructed with the TOMO3D software SIRT algorithm using 30 iterations (simultaneous iterative reconstructive technique) (Agulleiro and Fernandez, 2011). Semiautomatic segmentation of volumes was carried out with Amira software (Thermo Fisher Scientific), and volumes were represented with Chimera software (Pettersen et al., 2004).

### Epifluorescence microscopy of infected cells

HeLa cells producing stably GFP–SEPT6 were seeded in MatTek dishes and transfected with a plasmid encoding mCherry–LC3B (as described above). These cells were infected with *S. flexneri* AfaI for 30 min at 37°C and 5% CO_2_. Samples were then transferred to a temperature-controlled chamber (37°C and 5% CO_2_) and imaged in FluoroBrite medium (Life Technologies) supplied with 5% FCS, 4 mM L-glutamine (Sigma-Aldrich; #G7513) and 50 µg/ml gentamicin. Epifluorescence imaging was performed using an Axiovert Z1 microscope driven by ZEN Blue 2.3 software. Microscopy images were obtained as a *z*-stack image series taking 11 slices.

### *In vitro* reconstitution of *S. flexneri* – septin cages

*In vitro* reconstitution of septin cages was performed as described in Lobato-Márquez et al. (2021). An *S. flexneri* culture was grown 16 h in 50 ml conical polypropylene tubes containing 5 ml of M9-Tris (50 mM Tris-HCl pH 8, 50 mM KCl, 0.5 mM MgCl_2_, 0.1 mM CaCl_2_ and 1 mM MgSO_4_) salts supplemented with a mix of nutrients (45 µg/ml L-methionine, 20 µg/ml L-tryptophan, 12.5 µg/ml nicotinic acid, 10 µg/ml vitamin B1, 1% glucose, 0.5% casein hydrolysate, 0.1% fatty acid-free BSA), denoted M9-Tris-CAA, at 37°C with shaking at 200 rpm. The following day, bacterial cultures were diluted in 10 ml of fresh pre-warmed M9-Tris-CAA (1:100 v/v) in conical polypropylene tubes and cultured until an OD_600_ of 0.6 was reached. 1.2 ml of bacterial cultures were centrifuged in Low Protein Binding tubes (Thermo Fisher Scientific) at 800 ***g*** for 2 min at RT and the supernatant was removed. Purified recombinant septin hetero-oligomers (containing SEPT2, msGFP–SEPT6 and SEPT7) in septin storage buffer (50 mM Tris-HCl pH 8, 300 mM KCl, 5 mM MgCl_2_ and 5 mM DTT) were thawed on ice, diluted, and added to the *in vitro* reconstitution solution at a final concentration of 240 nM (yielding a final buffer composition of 50 mM Tris pH8, 50 mM KCl, 0.5 mM MgCl_2_ and 1 mM DTT). Low Protein Binding tubes containing the bacteria in the *in vitro* reconstitution solution were placed in opaque conical polypropylene tubes and incubated at 37°C with shaking at 220 rpm for 2 h. Following the *in vitro* reconstitution reaction, samples were immediately placed on ice. To remove unbound recombinant septin hetero-oligomers, samples were centrifuged at 800 ***g*** at 4°C for 1.5 min. Supernatant was carefully removed, and the bacterial pellet containing bound septins was resuspended in 300 µl of ice-chilled M9-Tris-CAA buffer and centrifuged at 800 ***g*** at 4°C for 2 min. This step was repeated one more time, to ensure removal of unbound septins, and pellets were finally resuspended in 100 µl of ice-chilled M9-Tris-CAA buffer. We placed 3 µl of this *in vitro* reconstitution reaction on EM grids that were plunge frozen and imaged by correlative light and cryo-SXT as described above.

### Immunostaining and confocal microscopy

Infected cells were washed three times with PBS pH 7.4 and fixed 15 min in 4% PFA (in PBS) at room temperature. Fixed cells were washed three times with PBS pH 7.4 and subsequently permeabilized 5 min with 0.1% Triton X-100 (in PBS). Cells were then washed three to six times in PBS and incubated for 1 h 30 min with primary anti-SEPT7, anti-FK2, anti-K63-Ub or anti-K48-Ub antibody diluted in PBS (1:100) supplemented with 0.1% Triton X-100 and 1% bovine serum albumin. Note that HeLa cells produce SEPT6 and SEPT7, which, together with SEPT2 and SEPT9, produce the hetero-oligomer SEPT2–SEPT6–SEPT7–SEPT9, which assembles into filaments and higher-order structures (Kim et al., 2011; Mostowy and Cossart, 2012). SEPT7 is essential for hetero-oligomer formation, and therefore SEPT7 staining represents the cellular distribution of the other septins (Martins et al., 2023). Cells were then washed three to six times in PBS and incubated 45 min with Alexa-Fluor-555-conjugated anti-rabbit-IgG or Alexa-Fluor-647-conjugated anti-rabbit-IgG secondary antibody diluted 0.1% Triton X-100 (in PBS). Cells were then washed three to six times in PBS and incubated with a solution of 0.1% Triton X-100 (in PBS) containing Hoechst 33342. Coverslips were placed on glass slides and samples were preserved with aqua polymount mounting medium (ID#18606, Polyscience).

Fluorescence microscopy was performed using a 63×/1.4 C-Plan Apo oil immersion lens on a Zeiss LSM 880 confocal microscope driven by ZEN Black software. Microscopy images were obtained as a *z*-stack image series taking 8–16 slices.

For Airyscan confocal live-cell imaging, MatTek dishes containing infected cells producing GFP–SEPT6 and mCherry–LC3B were placed on a temperature-regulated chamber (37°C and 5% CO_2_) and imaged using a 63×/1.4 C-Plan Apo oil immersion lens on a Zeiss LSM 880 confocal microscope driven by ZEN Black software. Microscopy images were obtained over time as a *z*-stack image series taking 8–16 slices using the Airyscan fast super-resolution (SR) mode.

### Image processing, quantification and statistical analysis

Confocal images of fixed samples were processed using Airyscan processing (Weiner filter) using ‘Auto Filter’ and ‘3D Processing’ options in ZEN Blue software. Confocal images of live samples were processed using Airyscan processing (Weiner filter) using ‘Auto Filter’ and ‘3D Processing’ options in ZEN Black software. Epifluorescence images were deconvolved using ZEN Blue.

Image quantifications were performed in Fiji. Where possible, fluorescence microscopy images were randomized using the macro for Fiji Filename_Randomizer.

Correlation between fluorescence microscopy and 2D soft X-ray images was done using the plugin ec-CLEM for Icy (Institut Pasteur, Paris, France).

Extraction of quantitative information related to mitochondrial anisotropy (deviation from spherical shape) on cryo-SXT volumes was performed using Amira software (Thermo Fisher Scientific). Extraction of quantitative information related to mitochondrial branch/rod length on epifluorescence images was performed using the Fiji plugin MiNa (Valente et al., 2017).

The analysis of K63 chains and LC3B associated to *S. flexneri* microdomains was performed in ImageJ. Airyscan confocal slices around the bacterial sagittal plane were maximum projected. Binary masks were then generated for each individual channel and combined. The regions of interests (ROIs) were fitted to an ellipse, and its long axis (identifying the bacterial poles) was determined mathematically (ImageJ macro 1). ROIs were reduced to 0.1–0.2 μm to fit the peak of maximum intensity of the microdomains and flattened between the points of intersection with the long axis. Finally, the intensity profile of the generated flattened image was quantified (ImageJ macro 2). Colocalization between microdomains was calculated defining that a microdomain has a normalized intensity ≥60% in the analyzed region. Custom ImageJ scripts (related to Fig. 4G–I) have been deposited in Github (https://github.com/ATLopezJimenez/macros_recruitment_to_bacteria).

Statistical analysis was performed in GraphPad Prism (v8.4, La Jolla, USA). Data represent the mean±standard error of the mean (s.e.m.) or median and interquartile range. Fold changes were calculated from each independent experiment and the mean±s.e.m. are given in the text. A Student's *t*-test (two-tailed), Mann–Whitney test or one-way ANOVA were used to test for statistical significance, with *P*<0.05 considered as significant. All statistical details including statistical tests, significance, value of the number of experimental replicates and bacterial cells quantified can be found in the figure legends.

All figures were designed using Adobe Illustrator CC 2018.

## Supplementary Material

10.1242/joces.261139_sup1 Supplementary information
