# Supplementary material for: Septins and K63 ubiquitin chains are present in separate bacterial microdomains during autophagy of entrapped Shigella
Source: J Cell Sci. 2023 Apr 13;136(7):jcs261139. doi: 10.1242/jcs.261139 (PMC10264824; doi:10.1242/jcs.261139)
Supplement: Supplementary information [file joces-136-261139-s1.pdf]

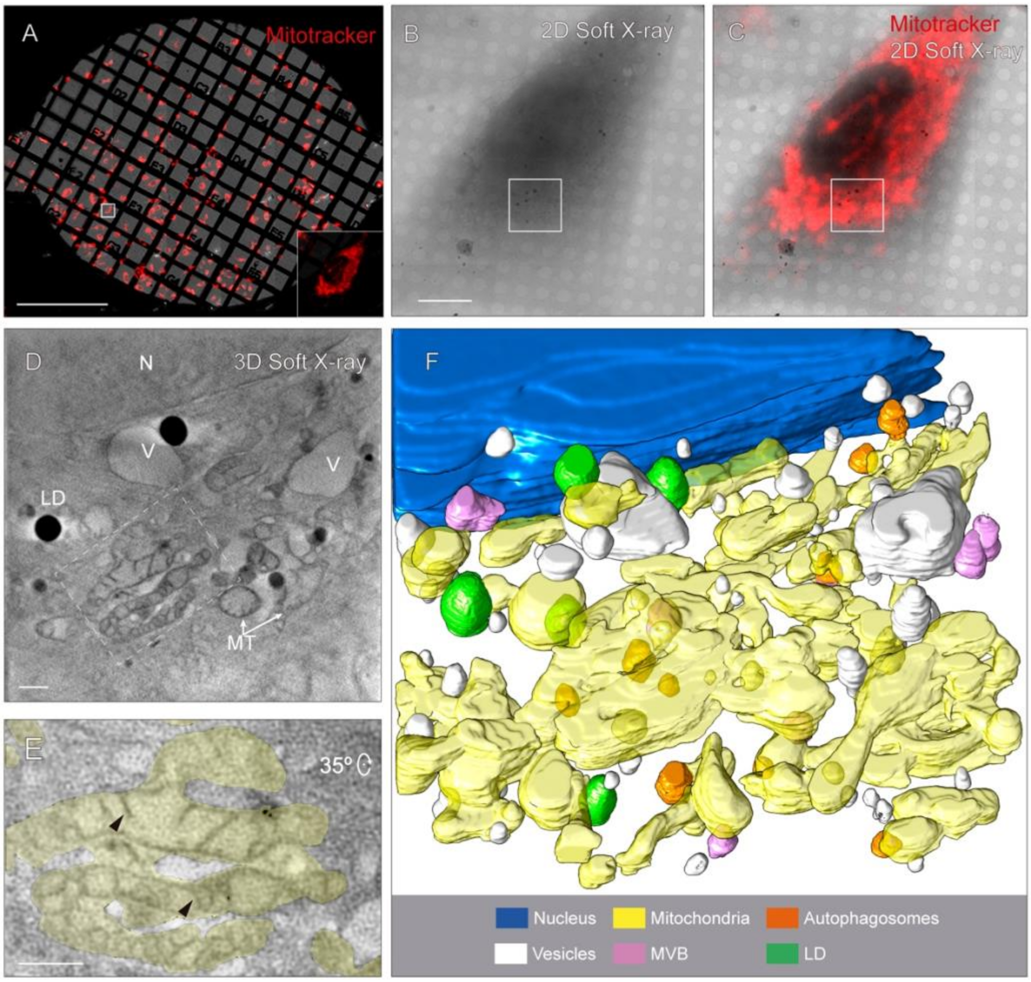

Fig. S1. Workflow for correlative light and cryo-SXT. To study the interplay between *S. flenxeri* septin cage entrapment and autophagy, we first developed a correlative light and cryo-SXT pipeline. We seeded human epithelial HeLa cells on Quantifoil (R2/2)-coated Au finder grids and plunge-freezed (vitrified) them in liquified ethane to preserve cellular structures. Before vitrification, cells were stained with Mitotracker Red, and samples were incubated with 100 nm gold fiducials to help with 3D reconstruction of tomograms. Vitrified samples were transferred to a Linkam stage and screened under liquid nitrogen temperature using a cryo-epifluorescence microscope to verify ice thickness, grid quality and locate areas of interest. Selected grids were transferred to a cryo-SXT microscope (operating under liquid nitrogen temperature) where grids were imaged by an on-line cryo-epifluorescence microscope (Fig. S1A). Scale bar, 500 μm. **(B)** Areas of interest (white square on (A) were subsequently imaged by soft X-rays, creating a mosaic that permitted identification of events of interest. Scale bar, 10 μm. **(C)** Correlating fluorescence and X-ray data permitted the identification of regions that could be imaged by X-ray tomography (e.g. white square on (B, C)). **(D)** After cryo-SXT was performed, raw tilt series were aligned and reconstructed. Images shown corresponds to a slice of 3.7 μm thickness. LD, lipid droplet; MT, mitochondria; N, nucleus; V, vesicles. Dotted square represents segmented mitochondria in (E). Scale bar,

Journal of Cell Science • Supplementary information

1 μm. **(E, F)** Reconstructed tomograms were segmented semi-automatically and rendered in 3D. In the inset of panel (E) it is shown a selected area from (D) depicting ultrastructural details of mitochondria. Mitochondrial crystae are indicated with black arrowheads. This approach enabled visualization of cell volumes at ~30 nm resolution by soft X-ray and its correlation with fluorescence data. From this pipeline, we could unambiguously identify cell nuclei, mitochondria and lipid droplets, as well as different types of vesicles and autophagosomes (see Fig. S2).

Journal of Cell Science • Supplementary information

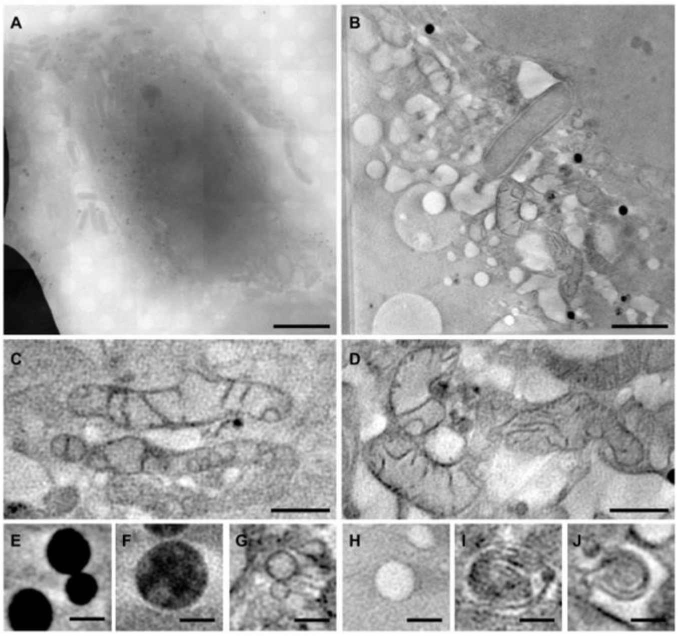

Fig. S2. Cryo-SXT atlas of HeLa cells in the context of *S. flexneri* infection. (A) Mosaic overview of a *S. flexneri*-infected HeLa cell. Scale bar, 10 μm. **(B-J)** Represent cell volumes containing different type of membrane-based host organelles or bacteria **(B)** Individual *S. flexneri* cell Scale bar, 2 μm. **(C, D)** Mitochondria appear as membranous cellular compartments with a lower X-ray absorbing inner part that contain membranous layers (cristae). Scale bar, 1 μm. (E) Lipid droplets were defined as spherical organelles with very high X-ray absorption due to the enrichment in lipids. Scale bar, 500 nm. **(F)** Multivesicular body, endocytic compartment that contain multiple vesicles inside, are seen as vesicles with variable X-ray absorption densities inside. Scale bar, 500 nm. **(G, H)** Vesicles are observed as endocytic compartments that contain a homogeneous inner X-ray absorption density. Scale bar, 500 nm. **(I, J)** Autophagolysosomes can be identified as endocytic compartments that show concentric membranous structures. Scale bar, 500 nm.

Journal of Cell Science • Supplementary information

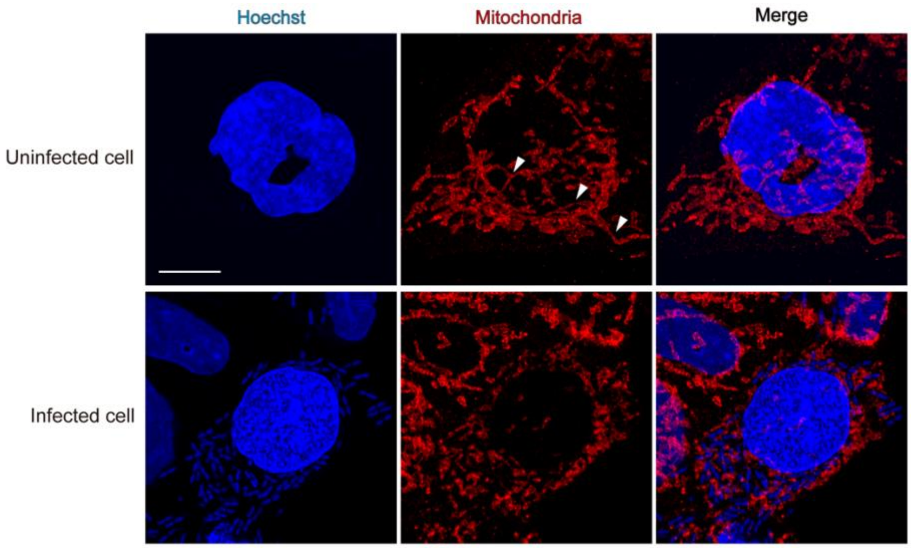

Fig. S3 related to Figure 1. Visualization of mitochondrial dynamics during *Shigella flexneri* infection by Airyscan confocal microscopy. Airyscan confocal images showing elongated mitochondria in the absence of infection (top panels white arrowheads) and mitochondrial fragmentation upon *S. flexneri* infection (bottom panels). Scale bar, 10 µm.

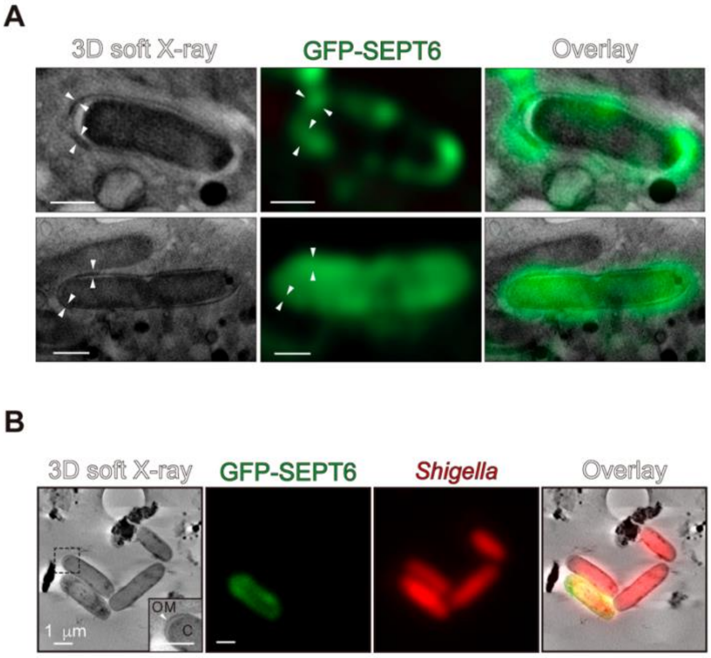

Fig. S4 related to Figure 2. Fluorescent signal of GFP-SEPT6 correlates to increased soft X-ray densities (arrowheads). (A) Additional examples related to Figure 2. Scale bar, 1 µm. **(B)** Representative example of a *S. flexneri* cell showing an extended periplasm at the bacterial cell pole, and next to it a separate bacterium entrapped in a septin cage reconstituted *in vitro* using purified septin complexes. Note that in this case there is no additional source of host membrane and proteins and thus septin cages cannot be visualized as X-ray dense structures. Scale bars, 1 µm.

Journal of Cell Science • Supplementary information

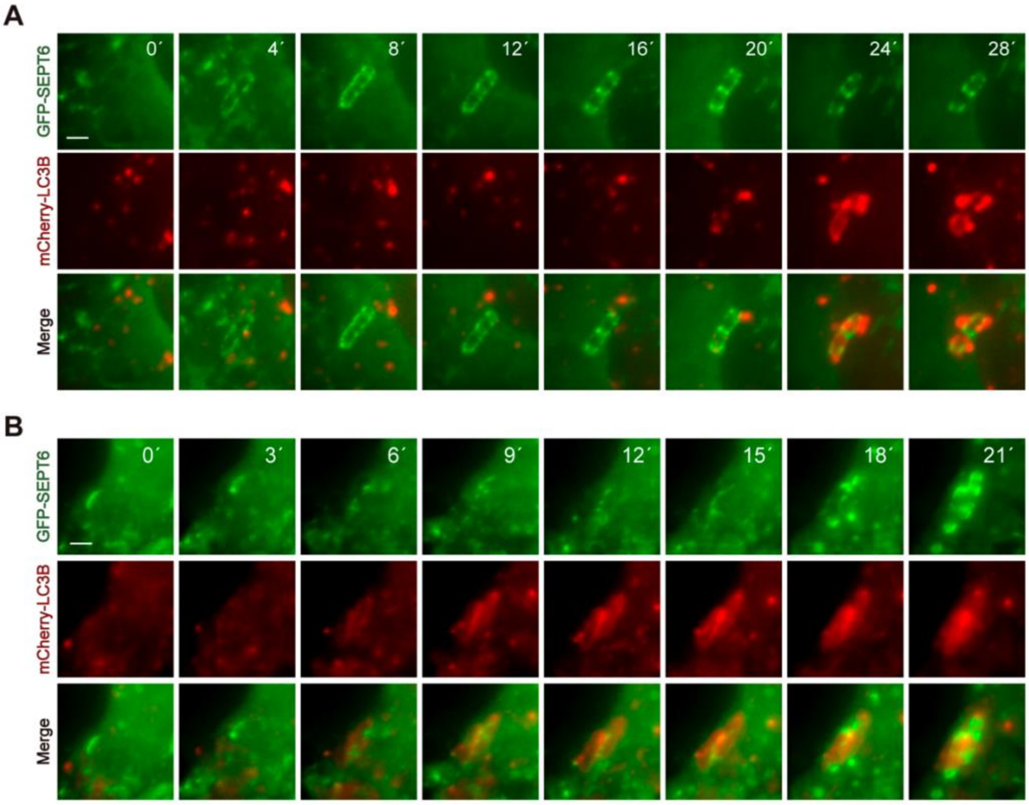

Fig. S5 related to Figure 3. Dynamics of *Shigella* septin cage entrapment and autophagy. (A) Epifluorescence time-lapse showing the entrapment of *S. flexneri* in a septin cage (labeled with GFP-SEPT6) followed by autophagosome formation (labeled with mCherry-LC3B). Scale bar, 2 µm. See also Movie S3. **(B)** Epifluorescence time-lapse showing the decoration of *S. flexneri* cells with mCherry-LC3B that are subsequently entrapped in 2 septin cages (labeled with GFP-SEPT6). Scale bar, 2 µm. See also Movie S4.

Journal of Cell Science • Supplementary information

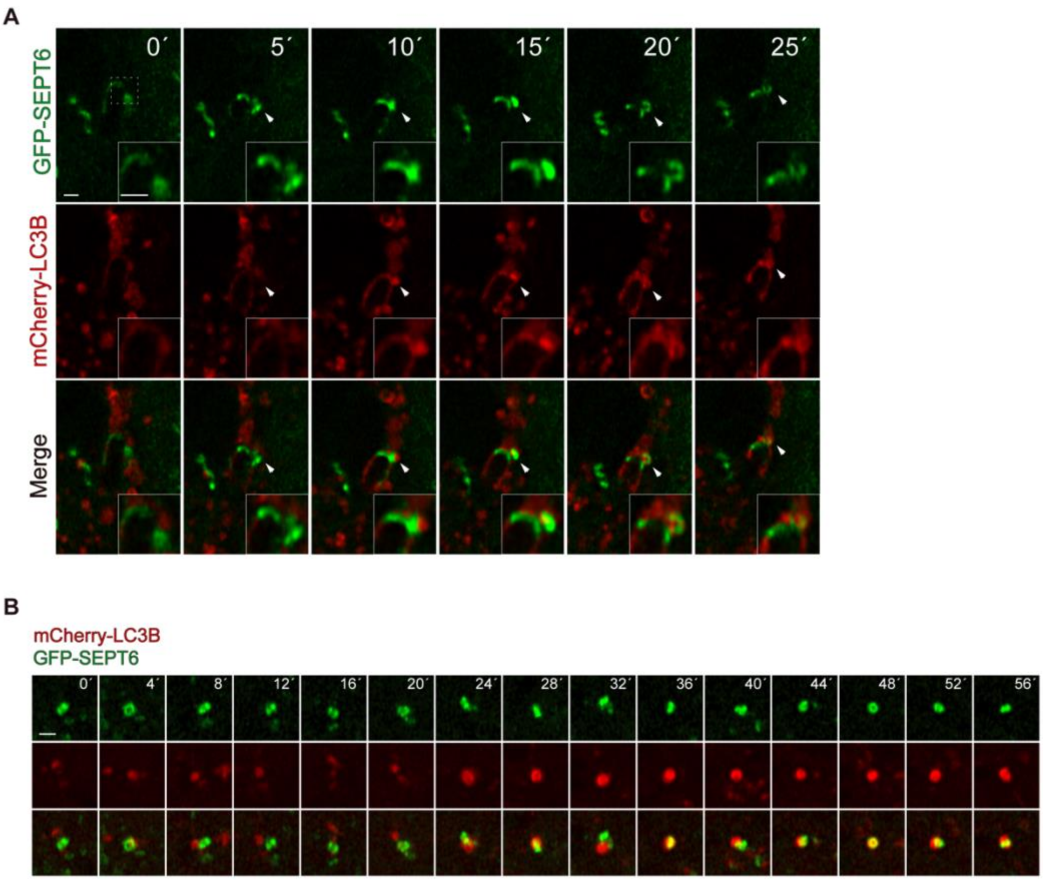

Fig. S6 related to Figure 3. Septins co-localize to autophagosomes as ring-like structures during infection of *S. flexneri*. (A) Airyscan fast super-resolution time-lapse showing a caged bacterium, where septins promote the fusion of LC3B to *S. flexneri*. White arrowheads point to the area where septins promote the recruitment of LC3B. See also Movie S5. Scale bar, 1 μm. **(B)** Airyscan fast super-resolution time-lapse showing a septin ring (labeled with GFP-SEPT6) bound to a forming autophagosome (labeled with mCherry-LC3B). See also Movie S6. Scale bar, 1 μm.

Journal of Cell Science • Supplementary information

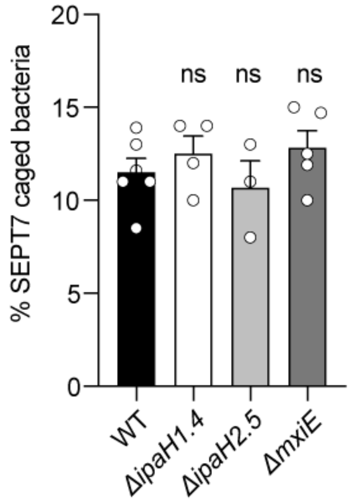

Fig. S7. *S. flexneri* E3-ubiquitin ligases do not impact septin cage entrapment. Percentage of *S. flexneri* M90T entrapped in SEPT7 cages in HeLa cells infected for 3h 40 min. Data represents the mean ± SEM from n = 1,466 (WT), n = 821 (Δ *ipaH1.4*), n = 851 (Δ*ipaH2.5*) and n = 1,089 (Δ*mxiE*) bacterial cells distributed in, at least, 3 independent experiments. ns, $p > 0.05$ by one-way ANOVA and Tukey´s post-test.

Journal of Cell Science • Supplementary information

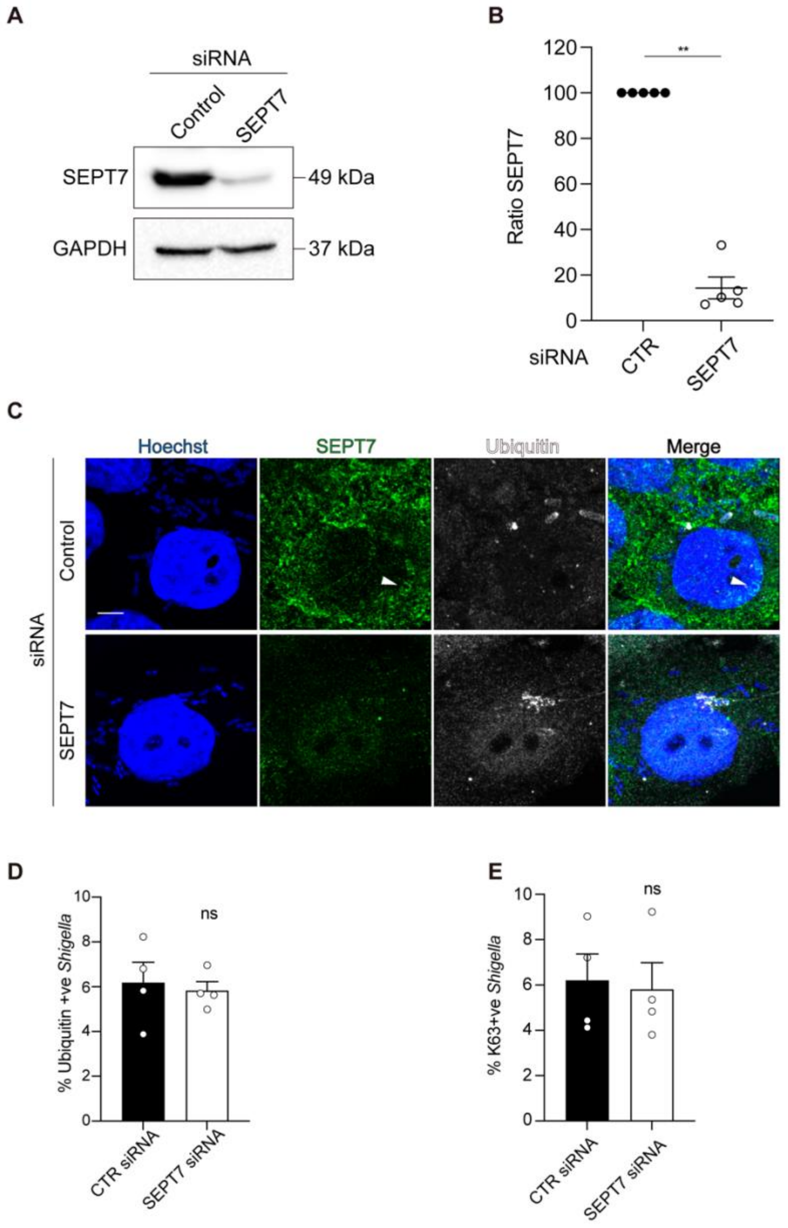

Fig. S8 related to Figure 4. Septins and K63 chains are independently recruited to *S. flexneri*. (A) Hela cells were treated with control or SEPT7 siRNA for 72h. Whole cell lysates were immunoblotted for SEPT7 to show the efficiency of depletion. GAPDH was used as loading control. **(B)** Western blot densitometry of 5 independent samples from (A) showing the absence of SEPT7 protein after siRNA treatment. **(C)** Airyscan confocal images showing *S. flexneri* positive with total (FK2) ubiquitin in HeLa cells infected for 3 h and treated with control (top) or SEPT7 (bottom) siRNA. White arrowhead, septin cage. Scale bar, 5 µm. **(D)** Quantification of *S. flexneri* decorated with total ubiquitin in the presence (CTR siRNA) or absence (SEPT7 siRNA) of SEPT7. Data represents the mean ± SEM from n = 1,578 (control siRNA) and n = 1,445 (SEPT7 siRNA) bacteria distributed in 4 independent experiments. ns, $p > 0.05$ by two-tailed Student´s t-test. **(E)** Quantification of *S. flexneri* decorated with K63 polyubiquitin in the presence (CTR siRNA) or absence (SEPT7 siRNA) of SEPT7. Data represents the mean ± SEM from n = 1,578 (control siRNA) and n = 1,445 (SEPT7 siRNA) bacteria distributed in 4 independent experiments. ns, $p > 0.05$ by two-tailed Student´s t-test.

Journal of Cell Science • Supplementary information

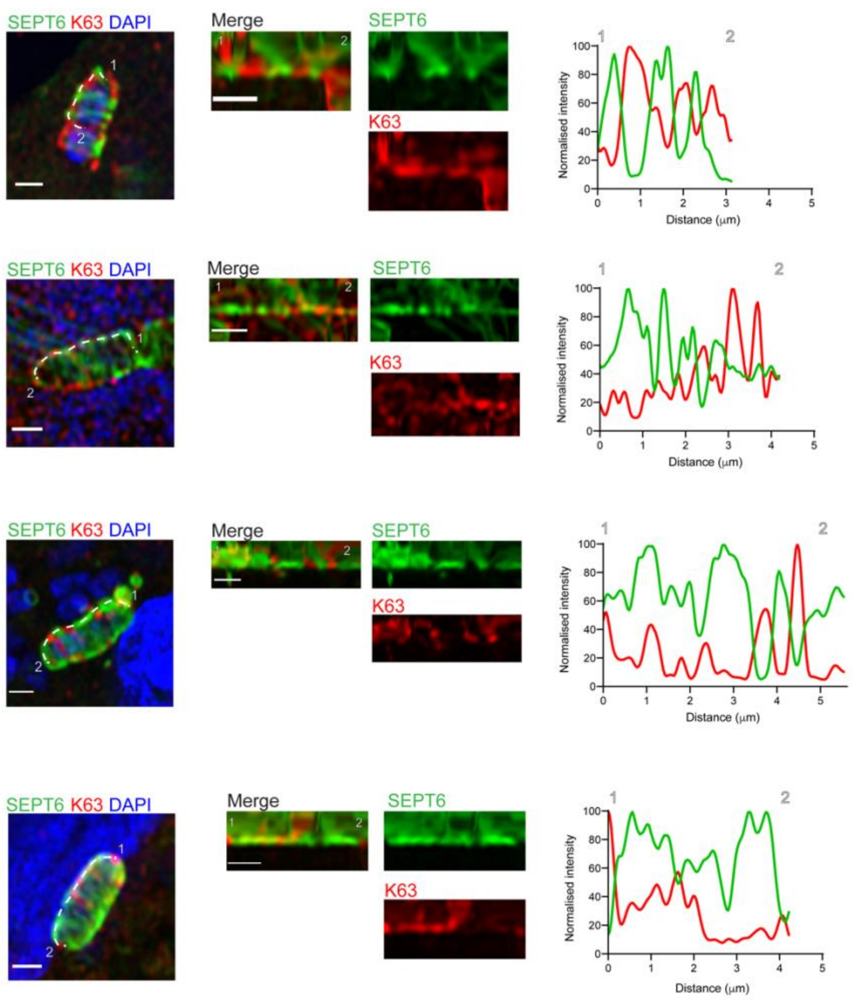

Fig. S9 related to Figure 4. Septins and K63 polyubiquitin form separate microdomains on the cell surface of *S. flexneri*. Airyscan confocal images showing the formation of separate microdomains of GFP-SEPT6 and K63 polyubiquitin on the surface of *S. flexneri* (left panels). Central panels represent the GFP-SEPT6 and K63 polyubiquitin microdomains of the septin cages from left panels. 1 and 2 mark the beginning and end of the dashed white line from left panels. On the right panels it is represented the fluorescence intensity profiles of GFP-SEPT6 and K63 polyubiquitin across the line from central panels. Scale bar, 1 μm.

Journal of Cell Science • Supplementary information

Table S1. Oligonucleotides used in this study.

Primer name	DNA sequence	Function
Fw-Del-IpaH1.4	CGGGCATGAAAAAAGCTACATCCGGTAGTAAACATTAT CAGTGGGTTATGGTGTAGGCTGGAGCTGCTTC	Deletion of *ipaH1.4* Forward primer
Rv-Del-IpaH1.4	GCTGAATTACCATTCTCAGGAATAACAGCAGACTCCTT CCCATAGACGGCTTCCATATGAATATCCTCCTTAGT	Deletion of *ipaH1.4* Reverse primer
IpaH1.4-Comp5	GGGCATGAAAAAAGCTACATCC	Confirmation of *ipaH1.4* deletion
IpaH1.4-Comp3	CACCATTATTCGAGTATAGGGAGAG	Confirmation of *ipaH1.4* deletion
Fw-Del-IpaH2.5	GGGTACGAATAAAGTAACGGGGGAAGCAATACCTGGA GAAAGAGTACAAAGTGTAGGCTGGAGCTGCTTC	Deletion of *ipaH2.5* Forward primer
Rv-Del-IpaH2.5	GTTCAGGCCAGTACCTCGTCAGTCAACTGACGGTAAAT CTGCTGTTCAGTCCATATGAATATCCTCCTTAGT	Deletion of *ipaH2.5* Reverse primer
IpaH2.5-Comp5	GGGAAGCAATACCTGGAGAAA	Confirmation of *ipaH2.5* deletion
IpaH2.5-Comp3	GGCCAGTACCTCGTCAGTCAA	Confirmation of *ipaH2.5* deletion
pKD4-Comp5	CCTGCGTGCAATCCATCTTGTTCA	Confirmation of km cassette insertion
pKD4-Comp3	GCATCGCCTTCTATCGCCTTCTTG	Confirmation of km cassette insertion

Journal of Cell Science • Supplementary information

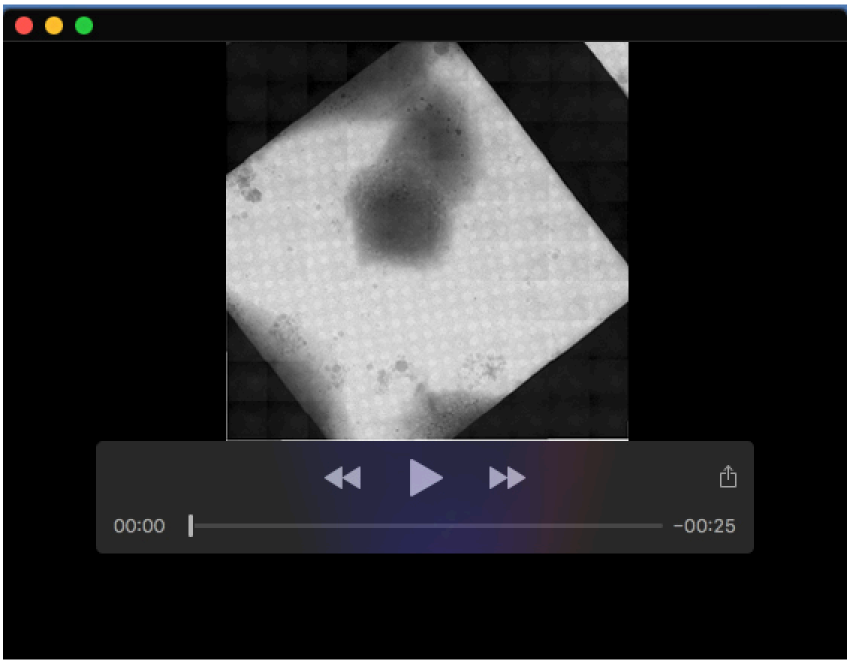

Movie 1. Correlative light and cryo-SXT movie related to Figure 2B.

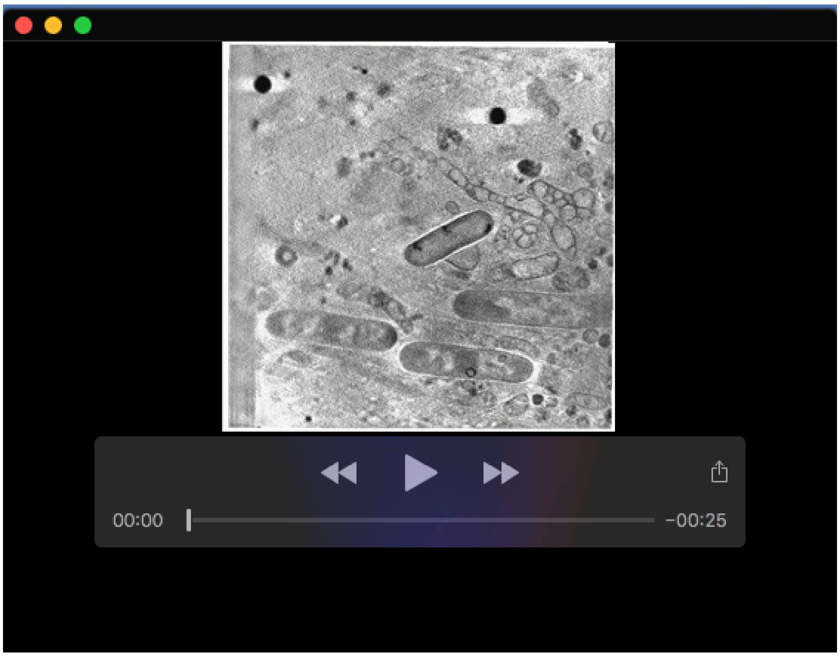

Movie 2. Correlative light and cryo-SXT movie related to Figure 3B.

Journal of Cell Science • Supplementary information

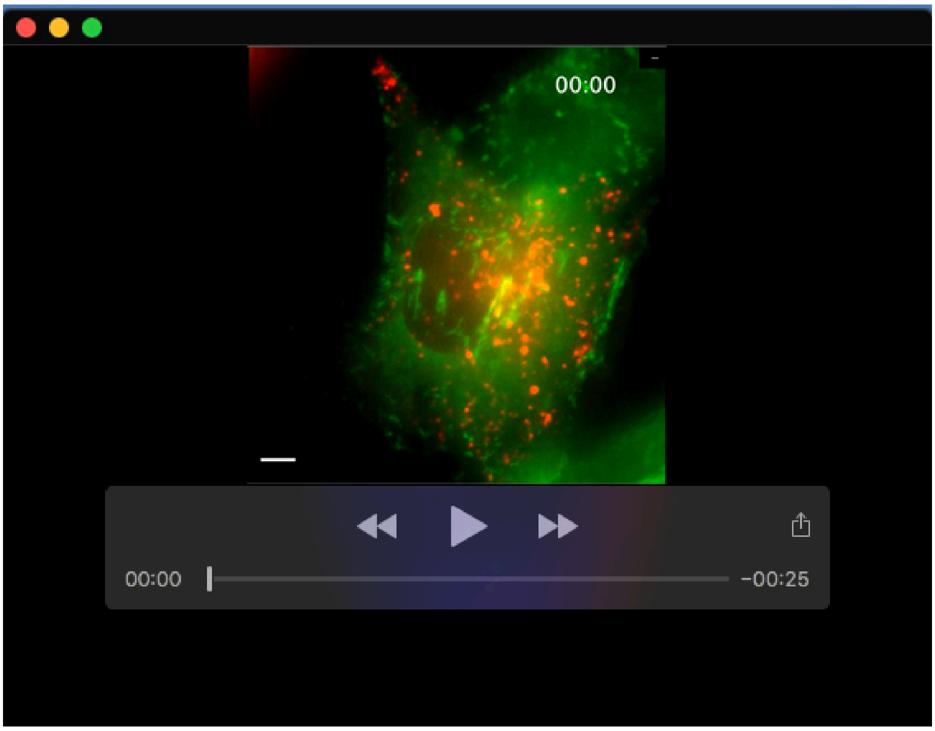

Movie 3. Time-lapse epifluorescence movie related to Fig. S5A. Images were acquired as 11 z-stacks every 4 min. Images were deconvoluted using ZEN Blue software and max projected. Scale bar, 5 µm.

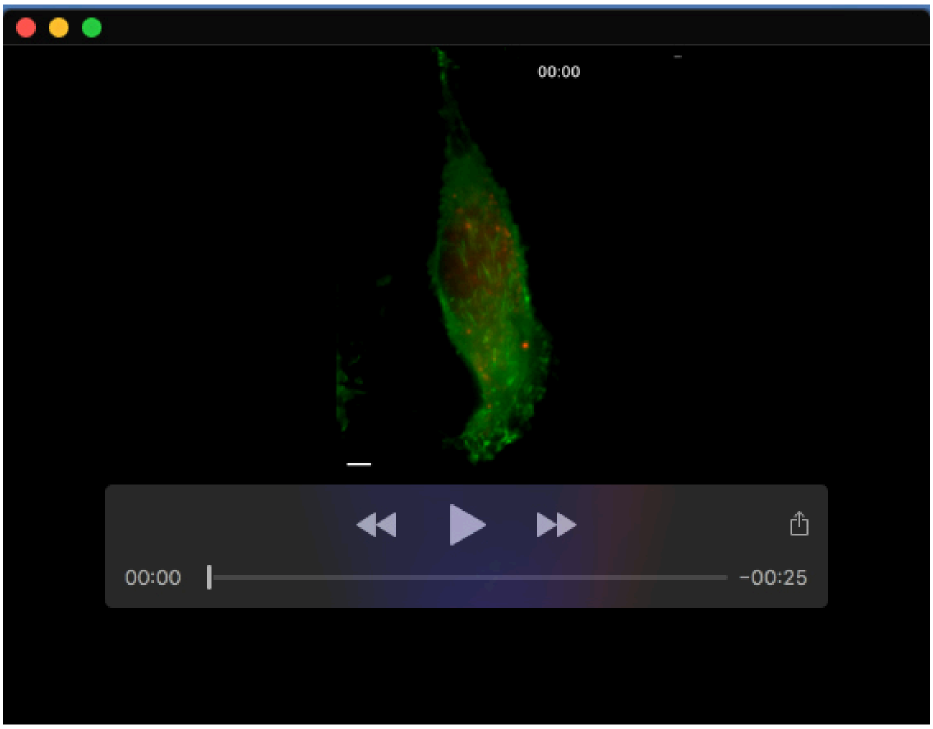

Movie 4. Time-lapse epifluorescence movie related to Fig. S5B. Images were acquired as 10 z-stacks every 3 min. Images were deconvoluted using ZEN Blue software and max projected. Scale bar, 5 µm.

Journal of Cell Science • Supplementary information

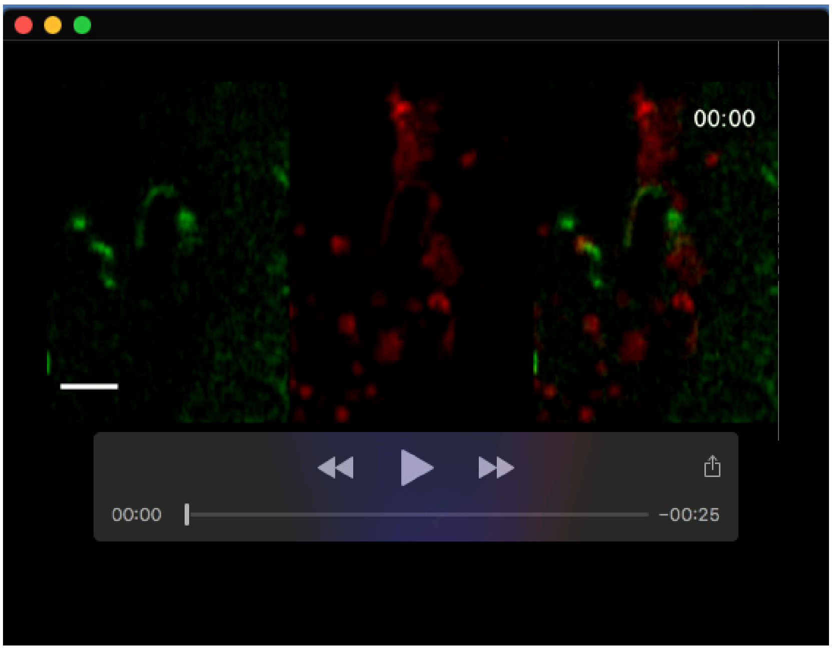

Movie 5. Time-lapse Airyscan confocal movie related to Fig. S6A. Images were acquired using Airyscan fast mode as 12 z-stacks every 5 min. Images were processed using “3D Airyscan processing” using ZEN Black software and max projected. White arrow indicates the interaction between GFP-SEPT6 and mCherry-LC3B mediating the recruitment of mCherry-LC3B to the *Shigella* septin cage. Scale bar, 1 μm.

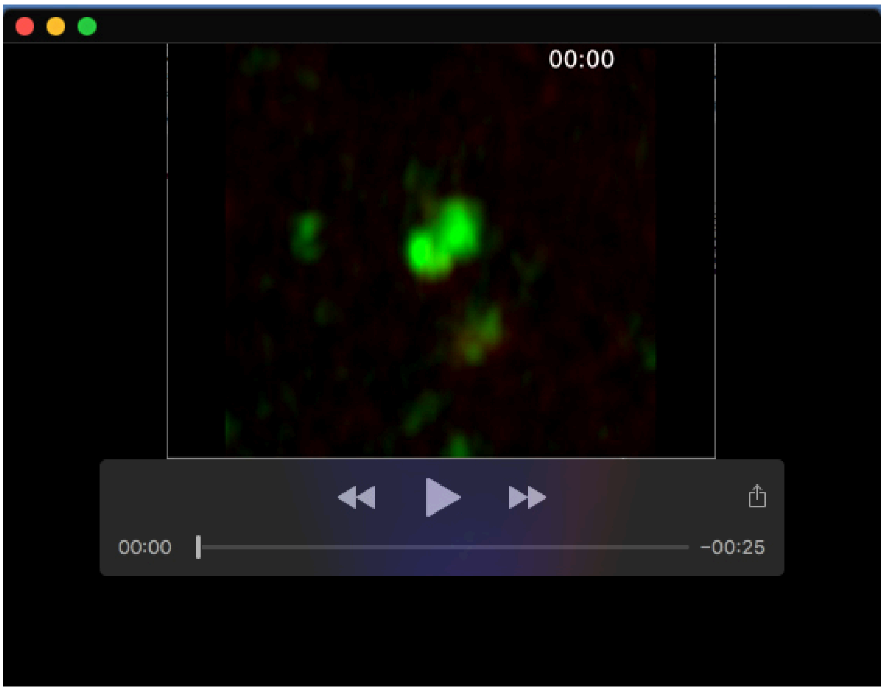

Movie 6. Time-lapse Airyscan confocal movie related to Fig. S6B. Images were acquired using Airyscan fast mode as 10 z-stacks every 4 min. Images were processed using “3D Airyscan processing” using ZEN Black software and max projected. Scale bar, 1 μm.
